# Super-Sentinel Chickens and Detection of Low-Pathogenicity Influenza Virus

**DOI:** 10.3201/eid1310.061552

**Published:** 2007-10

**Authors:** Philip I. Marcus, Theodore Girshick, Louis van der Heide, Margaret J. Sekellick

**Affiliations:** * University of Connecticut, Storrs, Connecticut, USA; † Charles River Specific Pathogen Free Avian Supplies, Storrs, Connecticut, USA

**Keywords:** chicken interferon, avian influenza, peroral adjuvant, influenza virus, sentinel chickens, dispatch

## Abstract

Chicken interferon-α administered perorally in drinking water acts on the oropharyngeal mucosal system as an adjuvant that causes chickens to rapidly seroconvert after natural infection by low-pathogenicity Influenza virus. These chickens, termed super sentinels, can serve as sensitive early detectors of clinically inapparent infections.

Early detection of low-pathogenicity type A influenza virus (LPAI) circulating among chickens is important for 3 reasons: 1) these are the most prevalent strains in nature and can cause substantial losses for commercial poultry producers (*1*), 2) these strains can contribute genetic material to high-pathogenicity type A influenza virus (HPAI) (*2*), and 3) the H5 and H7 LPAI strains can mutate to HPAI with catastrophic effects in birds, and with the potential for transmission to humans with lethal consequences (*3*). Kuiken et al. reported that an HPAI (H7N7) isolate was observed in February 2003 in the Netherlands, which most likely originated in free-living ducks and had evolved into a highly pathogenic variant after introduction into poultry farms (*4*). Although subsequent serologic screening of poultry showed that the H7 influenza virus had been affecting the Dutch poultry industry several months before the major epidemic, its presence had not been recognized (*4*). Our study addresses this problem by using a novel method that causes chickens to seroconvert under conditions in which LPAI would otherwise go undetected. This report shows that recombinant chicken interferon-α (rChIFN-α) (*5*) administered perorally in drinking water (*6*) acts as an adjuvant to produce a super-sentinel chicken that is a sensitive and early detector of clinically inapparent LPAI.

## The Study

In 2003, the first clue to an aberrant condition in a commercial flock of laying hens in Connecticut was signaled by a drop in feed consumption and then in egg production. It took 6–7 weeks from the time tracheal samples were sent to a diagnostic laboratory to confirm the diagnosis of LPAI (H7N2) infection at National Veterinary Services Laboratory (NVSL) (N. Adriatico, pers. comm.). One such isolate, A/CK/CT/72/2003(H7N2), was obtained from the US Department of Agriculture, NVSL, Ames, Iowa, and used throughout this study to determine whether the peroral administration of rChIFN-α under conditions found to ameliorate Newcastle disease (*6*), infectious bronchitis (*7*), and infectious bursal disease (*8*), would similarly affect avian influenza. We reasoned that if the spread of LPAI could be slowed or prevented, the probability of its mutating to HPAI would be proportionately reduced, thereby lowering the chances of transmission to humans. In the course of this study, we observed a strong adjuvant effect of rChIFN-α administered in drinking water under conditions of virus transmission that mimic natural infection in chickens. This led to the concept of the super-sentinel chicken described here.

Three-week-old specific-pathogen-free (SPF) white leghorns (Charles River Specific Pathogen Free Avian Supplies [SPFAS], Inc., Storrs, CT, USA) were tagged and divided into 2 groups of 10 chickens each. Two birds in each group were overtly infected intravenously or intranasally with 10^6^ infectious particles, measured as plaque-forming particles in primary chicken kidney cells (Charles River SPAFAS, Inc.). This strain of LPAI (H7N2) required a high inoculum to ensure infection (data not shown), comparable to that reported for another LPAI (H7N2) strain evaluated in SPF chickens (*9*). The 8 remaining cage mates in each group served as sentinel birds naturally subject to infection by the respiratory tract, ingestion of fecal material, or both. One group of birds received plain drinking water; the other group received drinking water that contained 2,000 U/mL rChIFN-α. The water was provided ad libitum and changed daily. Water consumption was the same in both groups, as determined from the amount remaining after a known volume was provided each day (data not shown). With a half-life of 3–5 days in water at room temperature (*6*), this concentration of interferon (IFN) delivered an average dose of ≈3 × 10^5^ U rChIFN-α/bird/day. Fourteen days post overt infection (dpi), the ChIFN-α-water was replaced with plain water for the remaining 14 days of the study. This dose of rChIFN-α was sufficient to ameliorate Newcastle disease (*6*).

**Figure 1:**
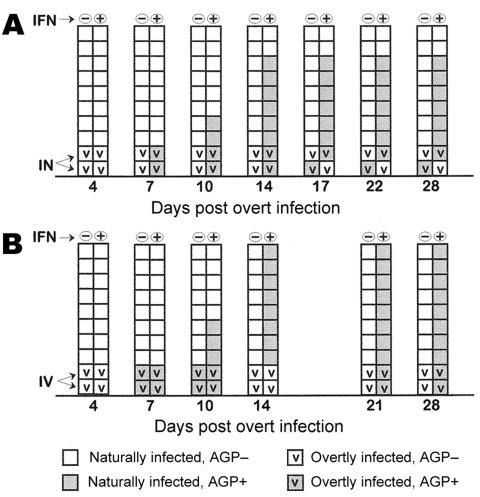
Seroconversion in specific-pathogen-free white leghorns after infection with influenza A/CK/CT/72/2003 (H7N2) as measured by agar gel precipitin (AGP) tests for avian influenza virus nucleoprotein and M1 antigens. Each box represents 1 chicken; (–), water; (+), water plus recombinant chicken interferon-α at 2,000 U/mL. IFN, interferon; IN, intranasal; IV, intravenous. A and B are independent trials. Serum samples were obtained at the times indicated on days post infection for overtly infected birds.

Following overt infection of 2 birds per cage, and the natural cross-infection of the 8 cage mates, serum samples were taken from each of the 10 birds at the intervals indicated in Figure 1. This figure shows data from 2 independent studies that used agar gel precipitin (AGP) tests to detect antibody against avian influenza virus nucleoprotein and M1 antigens. This qualitative test demonstrated that of the 16 naturally infected chickens given plain water, none seroconverted over the 28-day period they were exposed to the 2 infected cage mates. In marked contrast, of the 16 naturally infected chickens given water containing IFN, 14 were seropositive by 14 dpi and remained so during the 28-day test period.

**Figure 2:**
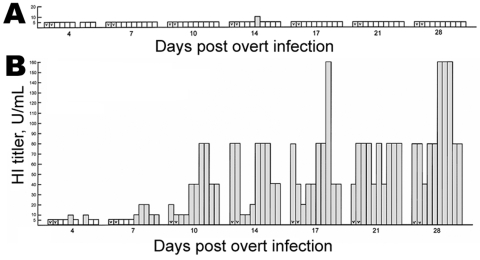
Seroconversion in sentinel specific-pathogen-free white leghorns after natural infection with influenza A/CK/CT/72/03 (H7N2) from overtly infected birds as quantified by hemagglutination inhibition (HI) tests for hemagglutinin (HA) antigen. The titer in HI U/mL is plotted as a function of days post overt infection of 2 birds in each group. The key is similar to that of Figure 1, except the assay is for HI. A, water only; B, water plus recombinant chicken interferon-α at 2,000 U/mL. Results of 1 trial are shown; 2 other trials gave similar results.

Figure 2 shows the number of seroconverted birds in a third study as quantified by hemagglutination inhibition (HI) titer (HI U/mL) of serum samples taken at the time intervals indicated as dpi. None of the 8 naturally infected birds given plain water seroconverted during the 28 days of the trial. In contrast, the 8 naturally infected chickens raised on IFN-water all seroconverted by 10 dpi (8/8), as did the overtly infected birds. Similar results were observed in 2 other trials. In all, 4 independent comparable trials were conducted, representing 2 AGP and 2 HI tests (Table 1). The marked contrast in the fraction of naturally infected birds that seroconverted on plain water and IFN-water is evident.

**Table 1:** Seroconversion in influenza A virus–infected 3-week-old chickens given water or water + interferon*

Infection type†	No. chickens		
	Water	Water + interferon‡	Total
Overtly infected	4/8§	8/8	16
Sentinel	2/32	31/31	63
Combined	6/40	39/39	79

*Represents 4 independent trials. †Overtly infected birds were mixed with uninfected sentinel cage mates, the latter to become infected naturally. ‡Recombinant chicken interferon-α at 2,000 U/mL (*5*). §No. positive birds/total no. receiving treatment, scored by agar gel precipitin or hemagglutination inhibition tests.

Although the sensitivity of LPAI to the action of IFN is well documented (*10*,*11*), rChIFN-α in the drinking water may have been exacerbating the infection, thereby leading to high levels of virus and antigen and high levels of seroconversion. This possibility was tested by using quantitative real-time reverse transcriptase (qRRT)–PCR to determine the amount of avian influenza virus in tracheal samples at 2, 4, and 10 dpi. Table 2 shows that within the error expected from testing individual chickens, the amount of infectious particle equivalents were not significantly different in birds given plain water or IFN-water. Thus, that more avian influenza virus antigen was produced in chickens that were given IFN-water is an unlikely explanation.

**Table 2:** Influenza A virus infectious particle equivalents (IPE) in tracheal swabs from sentinel chickens given water or water + interferon and infected naturally*

Day postinfection	Water (IPE/mL)	Water + interferon† (IPE/mL)
2	1,112 ± 1,353‡	760 ± 632
4	1,234 ± 764	463 ± 484
10	1,325 ± 398	2,113 ± 1,834

*Each cage contained 2 overtly infected birds and 8 cage mates as sentinels. Only sentinel birds are reported. Chickens were 3 weeks old at the start. †Recombinant chicken interferon-α in water at 2,000 U/mL (*5*). ‡Mean ± SD, n = 8. Qualitative real-time reverse transcriptase–PCR analysis with influenza A virus standard: IPE/mL.

## Conclusions

Although the role of IFN as an adjuvant when delivered perorally has been established in mammals (*12*), our data demonstrate for the first time, to our knowledge, that avian IFN administered in drinking water to naturally infected chickens lowers the threshold of antigen required to stimulate the adaptive immune response to an LPAI isolate. As a consequence, the action of perorally administered rChIFN-α in effect creates super-sentinel chickens that seroconvert in response to levels of antigen that would otherwise go undetected. Super-sentinel chickens would thus provide a novel means of detecting otherwise inapparent infections of LPAI, thereby buying time for its control or eradication.

We envision the introduction into a large flock of a number of small cages containing chickens in which IFN-water replaces plain water. These super-sentinel chickens will serve as sensitive early detectors of LPAI, like the proverbial canary used in mines to detect low levels of toxic gases. Because of the cross-reaction between chicken and turkey IFN-α (*5*,*13*), super-sentinel turkeys could likely be created in a similar manner. Super-sentinel birds could be replaced every month and possibly returned to production.

All strains of chickens tested, including those in the People’s Republic of China, have proved to be sensitive to the action of rChIFN-α (*14*). Genetically engineered production of rChIFN-α (*15*), treatment with it optimized for dose and duration, and its long half-life in water may make it economically feasible to convert many birds in a flock to super-sentinel status. It also may be prudent to set up super-sentinel birds in areas of high risk for avian influenza virus outbreaks, such as live-bird markets. Surveillance of other families of birds might be possible with species-specific IFN. Further studies are required to test these possibilities and the extent to which rChIFN-α functions as an adjuvant with other strains of avian influenza virus and chickens.

